# Comparison between the musician-specific seating position of high string bow players and their habitual seating position – a video raster stereographic study of the dorsal upper body posture

**DOI:** 10.1186/s12995-018-0217-6

**Published:** 2018-11-06

**Authors:** Daniela Ohlendorf, Jennifer Marx, Kathrin Clasen, Eileen M. Wanke, Stefan Kopp, David A. Groneberg, Stefanie Uibel

**Affiliations:** 1 Institute of Occupational Medicine, Social Medicine and Environmental Medicine, Goethe-University Frankfurt/Main, Theodor-Stern-Kai 7, Building 9A, 60590 Frankfurt/Main, Germany; 2 School of Dentistry, Department of Orthodontics, Goethe University Frankfurt/Main, Theodor-Stern-Kai 7, Building 9A, 60590 Frankfurt am Main, Germany

**Keywords:** Stringed bow player, Upper body posture, Three-dimensional back scan, Musician-specific seating position

## Abstract

**Background:**

Effects of playing high stringed bow instruments on the upper body posture have not been analysed so far. The instrument-specific seating position when playing in an orchestra is compared to the habitual seating position.

**Methods:**

Three dimensional back scans were performed in 13 professional violinists and viola players of a radio orchestra (8 f / 5 m). Trunk position in their habitual seating position and in the instrument- specific seating position imitating playing was compared. Statistical differences were calculated using Wilcoxon Matched Pairs Test with Bonferroni Holm correction.

**Results:**

Significant differences were found between the seated position with instrument and without (*p* < 0.001, 0.03, 0.02 or 0.01) in the spine (trunk length, sagittal trunk decline, lumbar bending angle, maximal rotation, standard deviation rotation, lumbar lordosis), the shoulder (scapula distance, scapula rotation, scapula angle right) and pelvis distance.

**Conclusions:**

Playing an instrument changes the static seating position by increased rotation of the spine and specific shoulder adaptations holding the instrument (left arm) and the bow (right arm), with minor effects on the pelvis. This forced position may result in chronic health effects. The method used in this study is an approach to better understand the involved muscular structures and possible resulting health damages.

## Background

Professional musicians frequently suffer from ailments due to forced postures demanded by a specific instrument. This has been shown as early as 1985 [1] and 1986 [2] in questionnaire-based surveys and is confirmed in a recent review [3]. Most importantly, the physical demands of playing a specific instrument facilitate musculoskeletal disorders, e.g. by asymmetrical and/or repetitive motions and forced postures [4]. Approximately 50% of professional musicians report these injuries [5, 6], with the highest incidence in high string bow players (viola and violin players) [7]. Künzel [8] reports that 70% of the movement apparatus problems are induced by the typical forced unfavourable playing posture combined with the restricted compensation possibilities.

These problems are especially pronounced in the cervical and lumbar spinal column and the upper extremities [5, 9] as shown in interview- and questionnaire-based studies. In string bow instrumentalists, during their training period, similar physical problems and intensive health impairments have been found in questionnaire studies, partly necessitating medical treatment [5, 10–15]. If not balanced by muscular activities the resulting posture deviations from the perpendicular line of the body may lead to malpostures and impaired playing ability [5]. These findings emphasize the importance of early preventive measures.

Early in the nineteenth century educational, physiotherapeutic, sports and musician interventions have been developed and implemented [5, 16, 17]. So far, their effectiveness in preventing or treating musician specific ailments has not been proven [5, 10]; modern techniques may be able to show effects of specific interventions for treatment and prevention.

For high string bow musicians, several publications deal with the effects of performing on the movement apparatus [18–23]. Video recordings or motion capture systems were used most often; they document instrument related movements [18–20, 24]. Kruta de Araújo et al. [21] used video detection of angular changes in body sections to record arm and head motions of playing violinists. Subsequent computer based evaluation was able to document posture differences. Due the camera position the video analysis is hampered by their two-dimensional recording planes (horizontal and vertical) [25]. Wasmer & Eickhoff [22] compared violinists in standing and seated playing position. In the playing motion while seated the upper trunk was more and the shoulder section less involved in the playing movements. Even the position of a violinist at the musical stand was important and resulted in different curvatures of the spinal column. Philipson et al. [26] used EMG to show higher activity in the *M. deltoideus*, M. biceps brachii and in the upper section of the M. trapezius in high bow instrumentalists reporting pain than those without pain. Levy et al. [27] compared violinists playing with or without shoulder support and found a reduced EMG activity in M. trapezius, M. sternocleidomastoideus and in the frontal part of *M. deltoideus* but without changes in M. biceps brachii. This is supported in an EMG-study of Berque & Gray [28] with an increased activity in the M. trapezius in pain free musicians, and by Wilkinson & Grimmer [29] quantifying tendon size by ultrasound.

No three-dimensional posture analysis of high string bow players has yet been published which included the differential analysis of all spinal sections as well as the shoulder and pelvis region. However, video raster stereography would allow this analysis. These data will allow the quantification of the strains on the musculoskeletal system and lead the therapy selection. In the present study the upper trunk position of high bow players is measured in their natural position, i.e. seated without an instrument, and compared to their typical playing seated position with their instrument. Using this approach, we will show and quantify the differences in posture within the shoulder, spine, and pelvis parameters.

## Material and methods

### Subjects

All 13 (8 w / 5 m) professional musicians playing bow instruments (violin / viola) of the “HR 3” radio orchestra in Frankfurt/Main (Germany) participated in this study. The mean age was 43.6 ± 9.9 years (range 32 to 59 years) and they have been playing their instrument for 36.9 ± 9.1 years, 6.12 days per week up to 4.19 h per day. All musicians were right–handed.

**Fig. 1:**
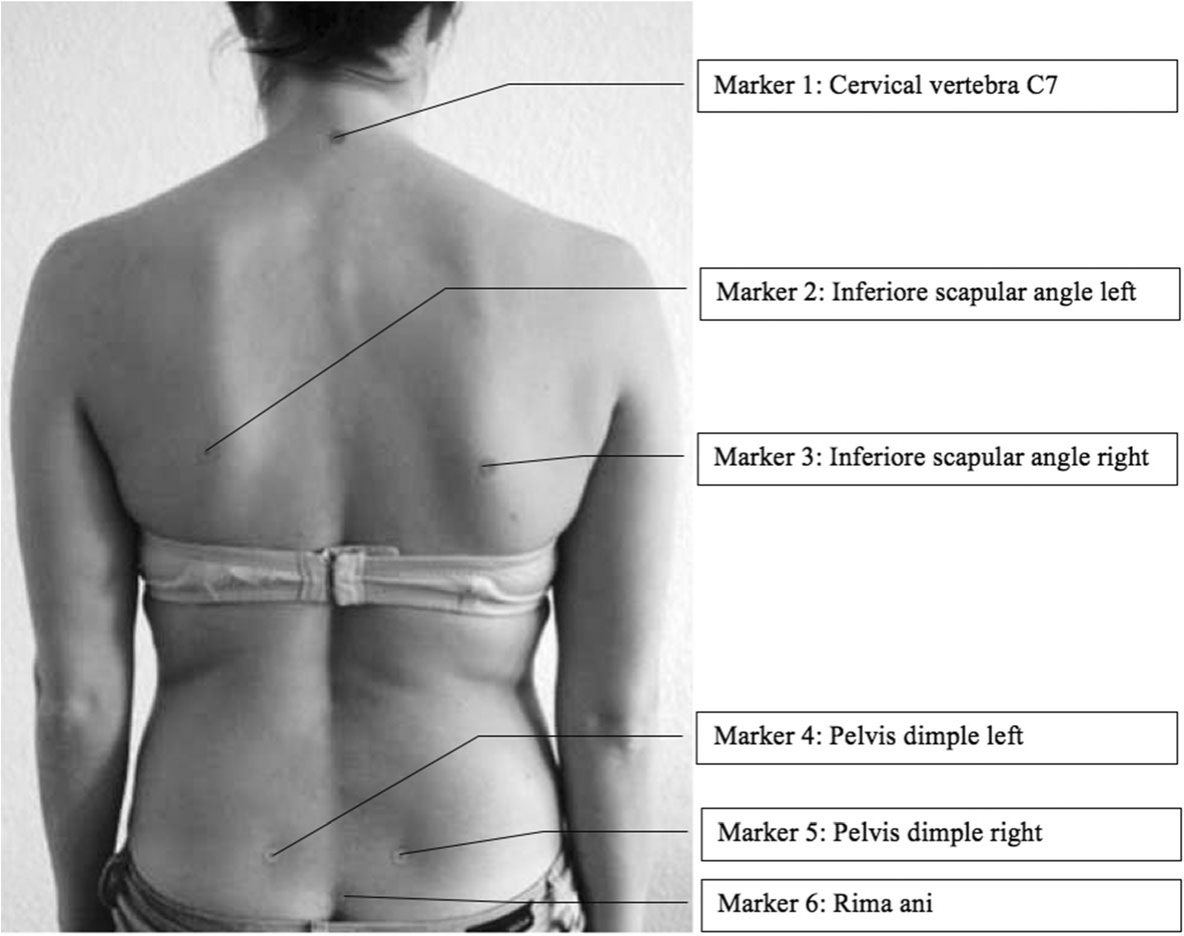
Location of the 6 marker

**Fig. 2:**
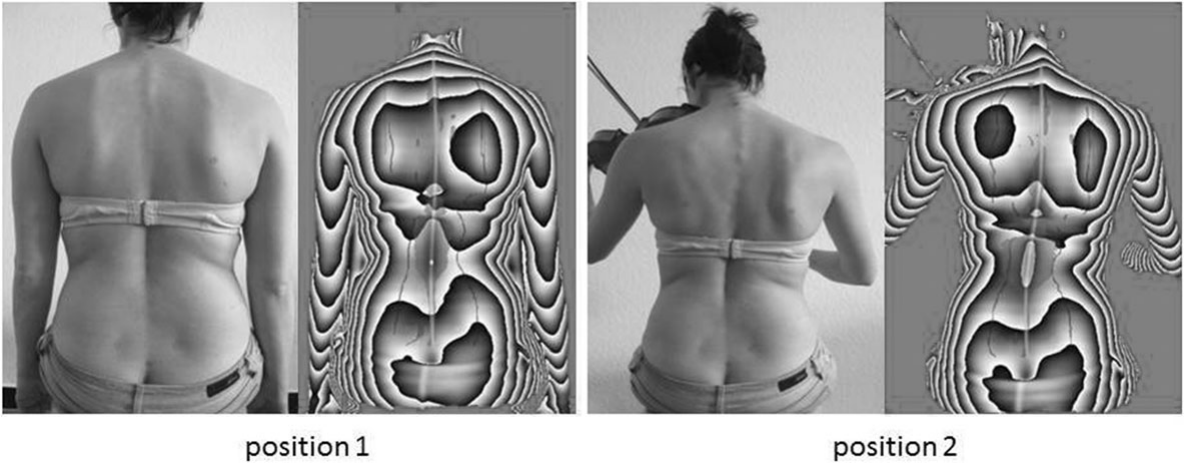
Group 1: Showing comparative positions 1 / 2

Exclusion criteria were acute trauma lesions, e.g. fractures, herniated discs and torn ligaments, surgery within the last six months, analgesics or muscle relaxant medication, genetic muscular or neurological diseases. All participants gave written informed consent to participate in this study, which was approved by the ethics committee of the Medical Faculty of the Goethe-University (Nr. 305/12), in accordance with the 1964 Helsinki Declaration and its later amendments. Furthermore, the individual of Figs. 1 and 2 in this manuscript has given written informed consent to publish these pictures.

### Measurement system

The back scanner “ABW-BodyMapper” (ABW GmbH; Frickenhausen / Germany) scanned the upper body posture by video raster stereography (Fig. 1) with 30 frames per second in the standard mode and a maximum of 50 frames per second. The camera resolution was 640–480 pixel over an area of 640 × 400 mm; depth resolution of the resulting image was 1 / 100 mm, lateral resolution < 1 mm as specified by the manufacturer. In repeated measurements using skin markers for reference positions the localization of a given point was accurate by an error margin of < 0.5 mm.

A sensitivity of 98%, a specificity of 84% and 13.9% false-positive values were published for the video raster stereographic survey [30]. Furthermore, raster stereography and radiological angle values presented excellent correlation according to Drerup (correlation coefficient 0.8 to 0.93; Drerup [31, 32]). Six self-adhesive markers were used to indicate anatomical landmarks as shown in Fig. 1. Drerup et al. [33] found this technique to be reliable with an intraindividual variation of 1 mm for the lumbar spine dimple.

This measurement system has been used in several studies [34–38]. It is a fast non-contact method to quantify body posture and it is suitable for measuring pathological postures like scoliosis, kyphosis or leg length discrepancies.

### Evaluation parameter

All parameters derived from the marker positions are listed in Table 1.

**Table 1:** List and description of back scan parameters

Spine parameter	
Trunk length D (TLD) (mm)	Spatial distance between the markers VP and DM
Trunk length S (TLS) (mm)	Spatial distance between the markers VP and SP
Sagittal trunk decline (STD) (°)	Inclination of the trunk length D marked line from the perpendicular to the sagittal plane. Tilt ventrally (negative values) = flexion Tilt dorsally (positive values) = extension
Frontal trunk decline (FTD) (°)	Inclination of the trunk length D marked line from the perpendicular to the frontal plane. Tilt anteriorly (negative values) = possible lordosis Tilt dorsally (positive values) = possible kyphosis
Axis decline (AD) (°)	Deviation of the line of the area marked by the trunk length D line of the 90° rotated distance DL-DR ➔decline between upper body and pelvis
Thoracic bending angle (TBA) (°)	Deviation of the distance VP - KA from the perpendicular
Lumbar bending angle (LBA) (°)	Deviation of the distance KA - LA from the perpendicular
Standard deviation lateral deviation (SDLD) (mm)	Root mean squared deviation of the median line of the distance VP - DM
Maximal lateral deviation (MLD) (mm)	Maximum deviation of the median line of the distance VP - DM Negative values = deviation to the left Positive values = deviation to the right
Standard deviation rotation (SDR) (°)	Root mean square deviation of surface rotation of the median line (torsion of the spinous processes of the spine)
Maximal rotation (MR) (°)	Maximum positive or negative surface rotation on the median line
Kyphosis angle (KA) (°)	In the sagittal plane measured angle between the upper inflection point of the spine at the thoracolumbar and VP inflection point IP; point of greatest negative surface decline
Lordosis angle (LA) (°)	Angle between the inflection point at DM and the thoracolumbar inflection point IP
Pelvis parameter	
Pelvis distance (PD) (mm)	Spatial distance between SIPS L and SIPS R.
Pelvis height (PH) (°)	Decline of the connecting line between SIPS L and SIPS R to the horizontal in the frontal plane in degrees
Pelvis height (mm)	Decline of the connecting line between SIPS L and SIPS R to the horizontal in the frontal plane in millimeter
Pelvis torsion (°)	Angle between the surface normal on the two dimples SIPS L and SIPS R Negative differential angle = Normal at point SIPS L is stronger upward as at point SIPS R Positive difference angle = Normal at point SIPS L is stronger downward as at point SIPS R.
Pelvis rotation (°)	Rotation of the distance SIPS L – SIPS R in the transversal plane
Shoulder parameter	
Scapular distance (SDI) (mm)	Distance between the left (AISL) and the lower right scapular angle (AISR).
Scapular height (SH) (°)	Height difference between the points AISL and AISR Positive value = AISR higher than AISL Negative value = AISR deeper than AISL
Scapular rotation (SR) (°)	Rotation of the distance DL-DR in the transversal plane
Scapular angle left (SAL) (°) / Scapula angle right (SAR) (°)	Best fit straight line on the shoulders to the horizontal. The center point of the regression line is set vertically above AISL / AISR. The greater the angle, the more caudally located the shoulder.

### Investigation protocol

All participants were seated on a regular musician chair and the upper body posture was measured with and without instrument in a randomized order. For measurements in the position without instrument, the markers were fixed on the bare back, and the participants took their preferred position (height, habitus, looking forward and hanging arms) with bared trunk. For measurements with their instrument the participants sat on their chair and played a medium pitch tone in the anticipated loudness “forte” with the middle of the bow. The left hand is placed on the 1st position of the fingerboard. They looked directly at the music stand, which is marked on the wall in front of them (Fig. 2). The posture was scanned for each position for 2 s, 3 scans were taken within 2 min and the parameters were calculated from the average of these data sets. In between the measurements the participants were allowed to relax for at least 2 min, all measurements including preparations lasted no longer than 15 min.

### Statistical evaluation

Statistical calculations were performed using the software BiAS 10.03 (Epsilon-Verlag, Darmstadt, Germany). Normal data distribution was tested by Kolmogoroff Smirnoff Lilliefors test. As the data sets were not normally distributed the Wilcoxon-Matched Pairs Test including a Bonferroni-Holm correction was used to test for significance. Significance is assumed at a difference level of 5%; significant differences are gives as: * = *p* < 0.05; ** = *p* < 0.01; *** = *p* < 0.001.

## Results

Posture differences in the upper body were quantified by comparing the parameters (Table 1), the results are presented in Table 2. The trunk length D is reduced from position 1 to position 2 with a mean of 497.65 ± 27.85 mm to 490.30 ± 25.22 mm (*p* < 0.001) in the sagittal plane. Concurrently, the trunk length S decreases (position 1: 551.29 ± 30.64 mm; position 2: 542.80 ± 28.24 mm; *p* ≤ 0.001). Sagittal trunk decline is characterized by a reduction from position 1 (− 7.67 ± 2.16°) to position 2 (− 3.73 ± 2.51°, *p* < 0.001), indicating a lower degree of leaning ventrally of the trunk. The lumbar lordosis increases as seen by the increasing lumbar bending angle from position 1 (6.29 ± 3.83°) to position 2 (8.53° ± 3.83°, *p* < 0.03). Holding the instrument results in an increased spinal rotation to the right; this is seen in the standard deviation rotation and maximal rotation from position 1 (SDR 4.25 ± 2.25°, MR 3.10 ± 7.47°) to position 2 (SDR 7.17 ± 4.84°, MR 12.42 ± 14.16°), with *p* < 0.001 for SDR and *p* < 0.01 for MR.

**Table 2:** Mean values (MV) and standard deviations (SD) of the all parameters with respect to position 1 and 2 as well as the calculated *p*-values of the Wilcoxon-Matched-Pairs-Test

Spinal parameters	Position 1		Position 2		*p*-value
Spinal parameters	MV	SD	MV	SD	*p*-value
Trunk length D (mm)	497.65	27.85	490.30	25.22	0.001***
Trunk length S (mm)	551.29	30.64	542.80	28.24	0.001***
Sagittal trunk decline (°)	−7.67	2.16	−3.73	2.51	0.001***
Frontal trunk decline (°)	0.53	1.03	1.34	1.99	0.13
Axis decline (°)	1.21	2.47	2.16	2.59	0.09
Thoracic bending angle (°)	13.53	4.42	14.14	3.33	0.45
Lumbar bending angle (°)	6.29	3.83	8.53	3.83	0.03*
Standard lateral deviation (mm)	3.45	2.37	4.06	3.31	0.64
Maximal lateral deviation (mm)	−3.24	6.31	−0.81	9.27	0.20
Standard deviation rotation (°)	4.25	2.25	7.17	4.84	0.001***
Maximal rotation (°)	3.10	7.47	12.42	14.16	0.01**
Kyphosis angle (°)	48.28	12.84	44.58	13.73	0.76
Lordosis angle (°)	32.89	24.58	24.79	11.10	1.00
Pelvis parameters					
Pelvis distance (mm)	92.46	7.14	91.32	7.48	0.02*
Pelvis height (°)	0.90	2.73	0.91	2.11	0.62
Pelvis height_2 (mm)	1.36	4.38	1.46	3.43	0.97
Pelvis torsion (°)	−0.08	2.23	0.81	3.77	0.42
Pelvis rotation (°)	0.87	3.51	2.41	5.63	0.31
Shoulder parameters					
Scapula distance (mm)	165.81	22.48	176.98	20.62	0.001***
Scapula height (mm)	0.26	8.35	−3.98	11.05	0.09
Scapula rotation (°)	2.87	3.18	5.76	5.82	0.02*
Scapula angle left (°)	29.05	5.55	40.55	19.16	0.13
Scapula angle right (°)	34,04	9,99	27,07	5,51	0.001***

Legend: * = *p* < 0.05; ** = *p* < 0.01; *** = *p* < 0.001

For the pelvis position a significant change has been found for the pelvis distance, which decreases from 92.46 ± 7.14 mm (position 1) to 91.32 ± 7.48 mm (position 2, *p* < 0.02) indicating an “inflare” motion of the os ilium.

**Fig. 3:**
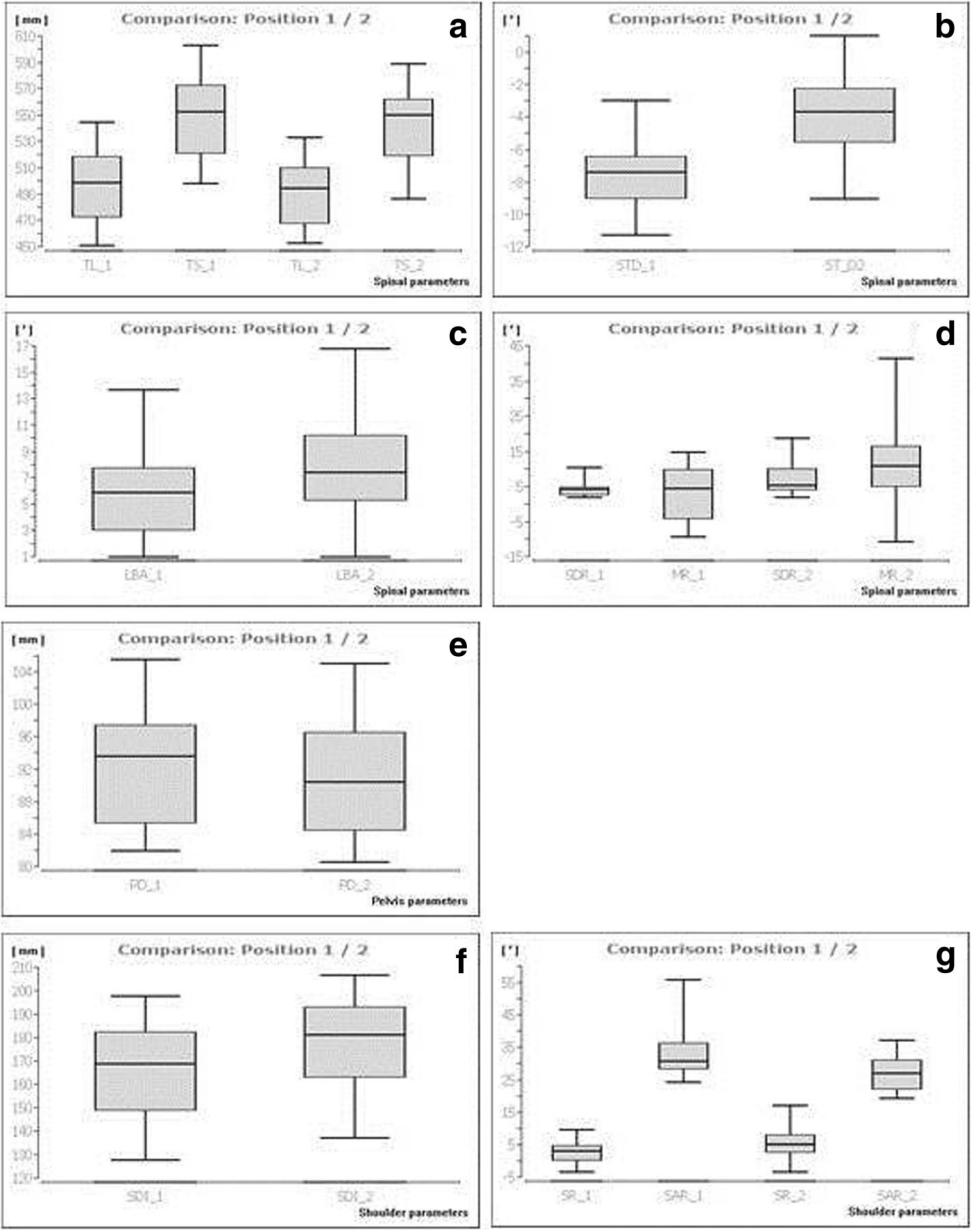
Upper body posture in high string bow instrument players. Legend: **a** trunk length S/D; **b** sagittal trunk decline; **c** lumbal bending angle; **d** standard deviation of the rotation/maximal rotation; **e** pelvis distance; **f** scapula distance; **g** scapula rotation; scapula angle right; for definition of the parameters see Table 1. Summarizes the significant differences induced when playing the instrument

As consequence, shoulder parameters were also effected by holding an instrument. The scapula distance increases from 165.81 mm ± 22.48 mm to 176.98 mm ± 20.62 mm (*p* < 0.001) due to abduced scapulae. The scapula rotation increased as well in position 2 from 2.87 ± 3.18° to 5.76 ± 5.82°, positioning the right angulus inferior scapulae further dorsally than the left one (*p* < 0.02) as caused by a left backward shoulder inclination and rotation). The right scapula angle decreased from 34.04 ± 9.99° to 27.07 ± 5.51° (*p* < 0.001), the positive values indicates a lower right shoulder while playing the instrument. In conclusion, differences in shoulder, spine and pelvis posture are detectable after adjusting to the instrument. All significances are shown graphically in Fig. 3.

## Discussion

The results show that playing a violin or viola induces major changes in the upper body posture. In order to quantify these changes, we compared the upper posture while playing with the participant’s habitual upper body posture, which has been chosen as the baseline position.

Playing the instrument increases spinal extension (TLD, TLS, STL), reduces the truncal inclination by spinal flexion (STL), increases lumbar lordosis (LBA) and rotated the upper body to the right (SR).

Habitual seating occurs spontaneously and results in an extension of the pelvis in the iliosacral joints with a corresponding decrease in lumbar lordosis [39]. This position centers the pelvis between the most anterior and the most posterior possible pelvis positions. Although it ideally distributes the static load it is difficult to sustain. However, this position is similar to the seated body position when playing a violin [39, 40].

Holding the instrument with the chin and positioning the left arm under the instrument causes a right sided dorsal upper body rotation. This rotation is increased by the requirement of looking at the centrally placed music stand in our setup, with the head rotated to the left to gaze at the imaginary music stand. This posture also changes the shoulder area as characterized by an abduced scapula (SD), a dorsal right shoulder (SR) and a lowered right shoulder with a cranially positioned scapula (SAR). This necessitates unfamiliar tonicity, especially for the right side and affects the M. levator scapulae and M. trapezius.

The changes in the shoulder area are caused by holding the instrument itself and the bow while playing. The shoulder of the instrument arm (left side) is held with the shoulder flexed, abduced, and externally rotated (Fig. 2).

In the pelvis area only the distance of the upper posterior iliacal crest markers is decreased and causes an “inflare” motion of the os ilium. If this also affects the tonicity it would impair the *M. quadratus* lumborum.

This study shows specific changes in the posture of violinists and viola players. They affect various sections of the upper body including the shoulder, pelvis and spine. Major alterations were observed in the spinal rotation and shoulder position and rotation with smaller effects in the spine length and pelvic distance verifying the working hypothesis. These changes can be explained by the specific demands of violin and viola playing. Corresponding findings were reported by Fishbein [2] and Kok [3]. Since the instrument-specific posture has to be held for many hours every day over many years they may result in chronic health effects.

In further studies, musicians of different playing levels could be differentiated with regard to playing related musculoskeletal disorder [41]. Here, however, the difficulty must be taken into account that professional musicians in particular are scheduled for rehearsals and concerts in such a way that participation in a scientific investigation is not top priority.

Since the physical constitution of the musicians cannot be changed and the postural demands cannot be altered it is advisable to optimize health prevention like as has already been done with exposure factors of farmers or fishermen [42–44]. One possibility would be to use the Cornell Musculoskeletal Discomfort Questionnaire to get a better insight of MSD [45].

## Conclusion

Professional musicians often complain about musculoskeletal problems from playing their instrument already early in their professional training [5, 10–15]. The method presented in this study illustrates the magnitude of the muscular changed in string bow players while playing their instrument. While playing the instrument spinal extension (TLD, TLS, STL) increases, the truncal inclination is reduced by spinal flexion (STL), lumbar lordosis (LBA) is increased and the upper body is rotated to the right (SR). Therefore, this information can be used to support physiotherapeutic treatment and to adjust prevention and/or rehabilitation. Due to the muscular changes that can be detected with this method, it should be discussed in the future whether a physical therapy should be a MUST HAVE for a professional orchestra in the future.

## Abbreviation

EMG: Elektromyography
