# Correction to: Comparison between the musician-specific seating position of high string bow players and their habitual seating position – a video raster stereographic study of the dorsal upper body posture

**DOI:** 10.1186/s12995-019-0229-x

**Published:** 2019-03-13

**Authors:** Daniela Ohlendorf, Jennifer Marx, Kathrin Clasen, Eileen M. Wanke, Stefan Kopp, David A. Groneberg, Stefanie Uibel

**Affiliations:** 1 Institute of Occupational, Social and Environmental Medicine, Goethe-University Frankfurt/Main, Theodor-Stern-Kai 7, 60590 Frankfurt/Main, Germany; 2 School of dentistry, Department of Orthodontics, Goethe University Frankfurt/Main, Theodor-Stern-Kai 7, 60590 Frankfurt am Main, Germany

**Correction to: Journal of Occupational Medicine and Toxicology (2018) 13:34.**

**https://doi.org/10.1186/s12995-018-0217-6**

After publication of the original article, the authors reported an error which needed to be corrected:

In the methodology section of the article [1], Fig. 1 and Fig. 2 have been mistakenly published. They exemplify the methodology of the back scan. The correct two figures are listed below. The legends remain the same.

**Fig. 1:**
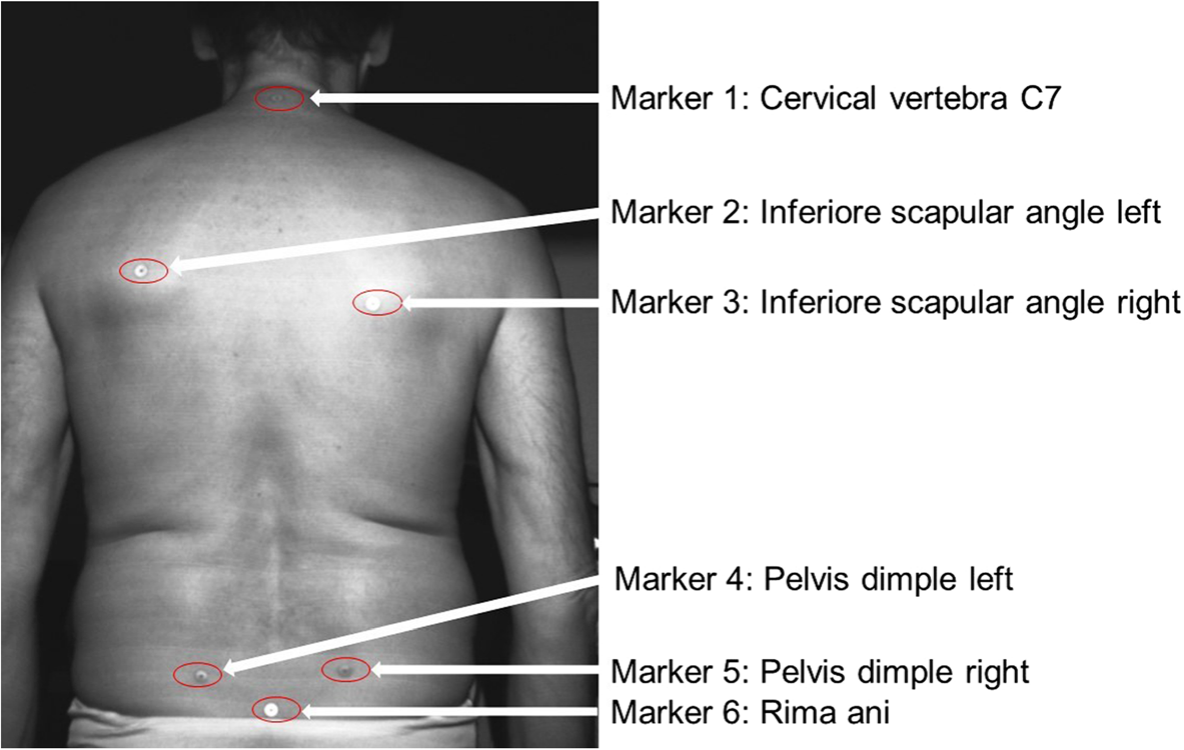
Location of the 6 marker

**Fig. 2:**
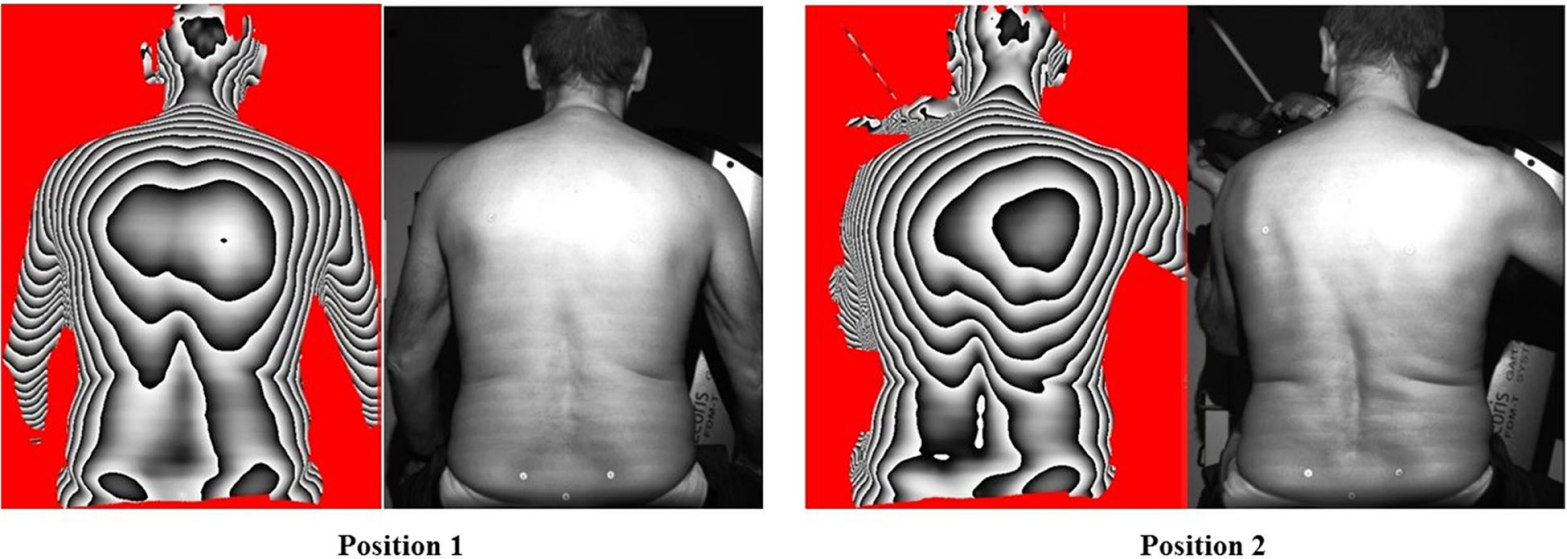
Group 1: Showing comparative positions 1 / 2
